# Perforated Ulcer of the Gastrojejunal Anastomosis and Concomitant Internal Hernia After One Anastomosis Gastric Bypass

**DOI:** 10.1007/s11695-023-06562-x

**Published:** 2023-03-29

**Authors:** Arnaud Liagre, Francesco Martini, Niccolo Petrucciani

**Affiliations:** 1 Clinique Des Cedres, Bariatric Surgery Unit, Ramsay Générale de Santé, Cornebarrieu, France; 2 Department of Medical and Surgical Sciences and Translational Medicine, Faculty of Medicine and Psychology, St Andrea Hospital, Sapienza University, Via Di Grottarossa 1035-9, 00139 Rome, Italy

**Keywords:** One anastomosis gastric bypass, Internal hernia, Anastomotic ulcer, Kehr’s drain

## Abstract

**Purpose:**

The management of concomitant complications after OAGB is challenging. We aim to show the surgical management of two concomitant complications after one anastomosis gastric bypass: internal hernia and anastomotic ulcer perforation.

**Materials and Methods:**

We present the case of a 32-year-old woman with BMI of 51 kg/m2, who underwent OAGB. Three years later, she presented with intense and brutal epigastric pain. She was a heavy smoker. Her weight and BMI were 75 kg and 26 kg/m^2^, respectively. Clinical examination showed generalized peritonitis, computed tomography showed pneumoperitoneum, diffuse peritoneal effusion, and rotation of the superior mesenteric vessels indicative of an internal hernia.

**Results:**

A generalized biliary peritonitis secondary to a perforated ulcer on the gastrojejunal anastomosis and internal hernia of the common loop into a large Petersen orifice were diagnosed. The internal hernia was reduced, and a perforation of the posterior surface of the gastrojejunal anastomosis was identified. Surgical treatment consisted in the placement of a Kehr’s drain into the perforation, closure of the Petersen orifice, and lavage-drainage of the peritoneal cavity. The postoperative course was uneventful, and she was discharged on postoperative day 12. The Kehr’s drain was removed 1 month after discharge.

**Conclusion:**

The combination of two different complications after OAGB can make the pre- and intra-operative judgment difficult and hamper the therapeutic approach. The initial reduction of the internal hernia made it possible to reduce the pressure in the surgical assembly and facilitated the treatment of the anastomotic perforation.

**Supplementary Information:**

The online version contains supplementary material available at 10.1007/s11695-023-06562-x.

## Introduction

The one anastomosis gastric bypass (OAGB) is a well-established bariatric procedure, with some theoretical advantages over the Roux-en-Y gastric bypass such as a lower rate of anastomotic ulcers and internal hernias [1]. Our tertiary referral center has a wide experience in the management of internal hernias, post-operative fistulas, and anastomotic ulcerative perforations, and we published the use of the Kehr’s drain in this setting [2–4]. In this multimedia article, we aim to describe an association of complications that can pose problems of pre- and per-operative management.

## Materials and Methods

We present the case of 32 years old patient who underwent OAGB with a 150 cm biliary loop in 2014 for morbid obesity, with a BMI of 51 kg/m2. She was a heavy smoker. Three years after the initial procedure, she presented with intense and brutal epigastric pain. Her weight and BMI were 75 kg and 26 kg/m2, respectively. Clinical examination showed generalized peritonitis, computed tomography showed pneumoperitoneum, diffuse peritoneal effusion, and rotation of the superior mesenteric vessels indicative of an internal hernia. Urgent laparoscopy was performed. No endoscopic option was possible due to the peritonitis.

## Results

A generalized biliary peritonitis secondary to a perforated ulcer on the “anterior surface” of the gastrojejunal anastomosis and internal hernia of the common loop into a large Petersen orifice were diagnosed. A careful and relatively easy reduction of the common loop from the cecum was performed. The OAGB was de-rotated by 180°, the perforation of the “anterior surface” was in fact a perforation of the posterior surface of the gastrojejunal anastomosis. Surgical treatment consisted in the placement of a Kehr’s drain in the perforation, closure of the Petersen orifice, and lavage-drainage of the peritoneal cavity. The postoperative course was uneventful and she was discharged on postoperative day 12. After CT scan control with oral contrast administration, the patient eats quickly with the T-tube in place, first soft food then solid.

The Kehr’s drain was removed 1 month after discharge.

## Discussion

The initial therapeutic tactic aimed to restore the normal anatomy of the bypass, first reducing the internal hernia before treating the ulcer perforation. The internal hernia can be asymptomatic because the opening of the Petersen defect is broad. In our experience, the rate of internal hernias requiring surgery is 3% after OAGB [3]. The risk of bowel ischemia is low. We do not close systematically the Petersen orifice during OAGB because we believe that the massive weight loss unravels a possible closure and closure may transform a large orifice into several small orifices with greater risk of strangulation. The small bowel is unrolled from the cecum and completely reduced from the orifice. A posterior perforation was treated with Kehr’s drain placement into the orifice. The use of the T-tube is one therapeutic option to close the perforated ulcer among others (direct suture, omental patch, etc.). We used this technique with excellent outcomes during peritonitis, surgery for chronic fistulas or perforated ulcers with very fragile tissues on their margins [1, 2, 4].

This latex drain causes inflammation and scar tissue formation. The Kehr’s drain was retired one month later with an excellent clinical outcome.

## Conclusion

The combination of two different complications after OAGB can make the therapeutic approach challenging. The initial reduction of the internal hernia made it possible to reduce the pressure in the surgical assembly and facilitated the treatment of the anastomotic perforation.

## Supplementary Information

Below is the link to the electronic supplementary material.

Supplementary file1 (MP4 169328 KB)
